# Molecular Screening Strategy to Identify a Non-invasive Delivery Mechanism for the Treatment of Middle Ear Disorders

**DOI:** 10.3389/fmed.2020.503819

**Published:** 2020-12-18

**Authors:** Arwa Kurabi, Molly Cooper, Meghan Spriggs, Yuge Xu, Daniel Schaerer, Allen F. Ryan

**Affiliations:** ^1^Department of Surgery/Otolaryngology, UCSD School of Medicine, San Diego, CA, United States; ^2^Department of Neurosciences, UCSD School of Medicine, San Diego, CA, United States; ^3^San Diego VA Healthcare System, San Diego, CA, United States

**Keywords:** phage display, peptide library, targeted therapy, middle ear, delivery, tympanic membrane (TM)

## Abstract

Middle ear ailments include a broad range of pathological conditions. Otitis media is the leading middle ear disease of childhood, which incurs significant health care resources in developed countries and, in developing countries, causes significant mortality and morbidity. Recurrent and chronic infections of the middle ear lead to the prolonged presence of inflammatory factors and cellular infiltrates resulting in temporary hearing loss. However, long-term alteration of the middle ear space can pose the risk of permanent damage to the delicate ear structures and cause tissue remodeling. While the etiopathogenesis of middle ear diseases is multifactorial, targeting the biological mechanisms and molecular networks that drive disease development is critical. Yet, a pivotal step in realizing the potential of molecular therapies is the development of methods for local drug delivery, since systemic application risks side effects. Utilizing bacteriophage display in the rat, we discovered rare peptides that are able to transit the intact tympanic membrane from the external canal to the middle ear cavity by an active process. An *in vitro* assay demonstrated that transport occurs across the tympanic membranes of humans and that the peptides cross the membrane independent of phage. Transport of phage, which is ~900 nm in length, suggests that these peptides could non-invasively deliver drug packages or gene therapy vectors into the middle ear.

## Introduction

### Diseases of the Middle Ear

Otitis media (OM) is a serious disease of the middle ear (ME). It is the most common reason for physician visits and surgery in children, resulting in a substantial medical and societal burden in developed countries (1, 2). However, in developing nations where access to more advanced medical care is limited, OM and related intracranial complications cause an estimated 28,000 annual deaths. Moreover, undertreated OM is estimated to be responsible for one half of the world's burden on serious hearing loss, with ~225 million cases (3).

The most common bacterial organisms causing OM are *Streptococcus pneumoniae*, non-typeable *Haemophilus influenzae* (NTHi), and *Moraxella catarrhalis* (4). Antibiotics are recommended for OM treatment in children under 2 and for complicated OM in older individuals (5). However, treatment of OM with antibiotics is controversial due to rise in antibiotic resistance (6, 7). Moreover, in up to 15% of children, little or no antibiotic may reach the ME (8). Local delivery of antibiotic to the ME can alleviate these issues but requires breaching the tympanic membrane (TM) by a trained surgeon. Children with chronic OM are often candidates for tympanostomy tube insertion (9), but this typically requires general anesthesia.

The reasons behind the persistence of ME infections is a subject of great interest in order to improve prevention and treatment outcomes. The identification of genetic predisposition factors and disease-related pathway has been the focus of research efforts for many years. Current evidence implicates many genetic factors and host immunodeficiencies in the predilection for recurrent OM (10). Researchers using immunodeficient mouse models demonstrated that innate immunity and its related pathways play a significant role in OM pathogenesis and recovery (11).

Cholesteatoma is a potentially destructive, progressive disease in which keratinocytes invade the ME and grow, often aggressively. Cholesteatomas are osteoclastic and can destroy the structures of the middle and inner ears and, in extreme cases, progress intracranially (12). Treatment involves surgical removal of the cholesteatoma mass. However, it is often impossible to remove all of the cholesteatoma tissue, and remnants can continue to grow leading to recurrence and further surgery (13). Drugs to inhibit tissue growth and osteoclast function have been proposed as potential treatments (14) but have not yet been applied to the disease.

The development of cholesteatoma receives contributions from several biological processes related to cell migration, proliferation, extracellular matrix deposition, and tissue remodeling (15). While cholesteatoma can be congenital or acquired, congenital only accounts for 3% of the cases (13). The mechanisms of cholesteatoma pathogenesis are subjects of debate. Nonetheless, recent biomolecular advances and DNA analyses of the cholesteatomatus masses revealed that the cholesteatoma epithelium has high proliferative activity (16) and higher expression of many inflammatory receptors and mediators such as TLR4, TLR2, NOD2, TNFα, and IL1β (17, 18).

In both OM and cholesteatoma, local delivery of drugs has the potential to be beneficial. Drug delivery that did not require penetration of the TM would be especially useful, as it could reduce the need for surgery and general anesthesia in children, and could also be used in situations in which access to ENT surgeons is limited. Moreover, gene therapy could rescue patients with genetic mutations causing predisposition to such diseases. Such therapies would benefit from local delivery to the ME, since these molecules have a broad target range in many tissues and organs that would preclude systemic delivery. In this review, we describe our most recent efforts to discover and validate peptides that can cross the intact TM while carrying large cargo for delivery into the ME as a therapeutic strategy. This would include drugs, drug packages such as nanoparticles, or gene therapy.

### Clinical Applications of Phage Display

Bacteriophages are viruses that infect bacteria, replicating in large numbers followed by phage release and continued infection. In phage display, each virus is genetically engineered to express different peptide sequences fused to one of its extracellular proteins (19, 20). Phage display has been shown to be useful as a high throughput-screening tool for the identification of peptides with specific targets (21). It has also been applied in the development of clinically novel therapeutics and diagnostic agents (22). Phage particles can be used as the therapeutic agent themselves. For example, phage therapy was successfully used to treat a multidrug-resistant bacterial infection by intravenous injection of bacteriophage cocktail that targeted and consumed that specific bacterial strain (23). In another example peptide phage was genetically engineered and utilized to deliver DNA encoding bactericidal toxin proteins (24). Hence, peptide-based therapeutics have attracted a significant level of interest in the drug discovery and development industry to enhance tissue targeting and delivery of therapeutic cargo.

We hypothesized that the TM might harbor the capacity to actively transport substances from the external canal into the ME (Supplementary Figure 1). Moreover, we posited that the most likely candidates for such transport would be biological molecules recognized by cellular receptors on the external surface of the TM. To address this hypothesis, we utilized bacteriophage display to screen large numbers of peptide sequences for the ability to mediate trans-TM transport (25). For our study, we harnessed this rabid combinatorial power to screen a library expressing peptides that are 12 amino acids in length that juxtaposed to the N-terminal, free end of the pIII protein of M13 bacteriophage. Five copies of this filamentous, extracellular protein per phage particle mediate the recognition of and binding to the *E. coli* hosts of the phage, and the expressed peptides are thus well-positioned to contact cells.

### Phage Display Reveals Rare Peptides That Can Cross the Intact TM

We screened a phage library from New-England Biolabs in which 10 phages expressing each of 10^9^ random peptides, for a total of 10^10^ phages, were applied onto the TM in NTHi-infected rats for 1 h. Two *in vivo* biopanning strategies were employed to screen the library, using rats whose MEs had been infected 48 h previously. ME infection induced by NTHi in the rat model was used during the phage selection process since NTHi is a common pathogen isolated during ME infections, and our goal is to provide a preclinical basis for clinically applicable pharmacotherapy.

**Figure 1:**
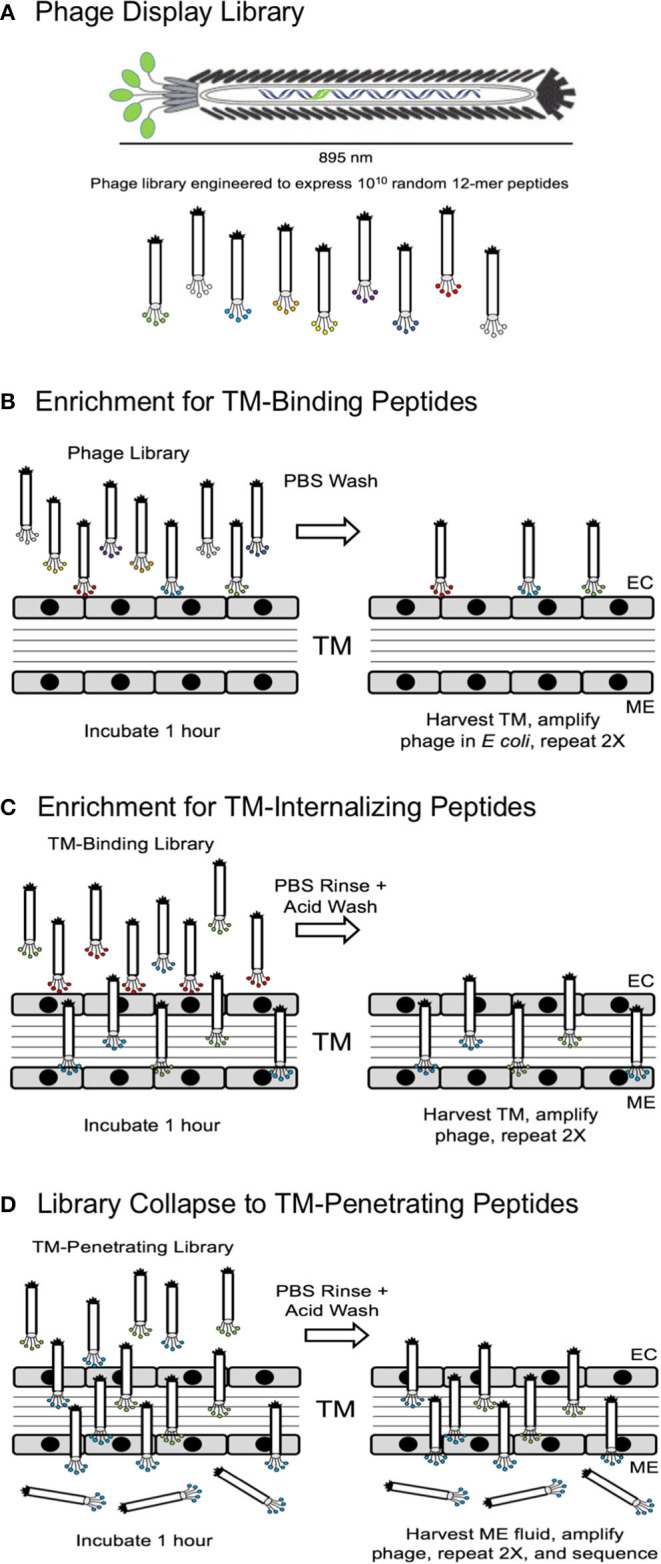
**(A)** Schematic representation of a phage display library, showing expression of random peptides on the free end of M13 bacteriophage pIII protein. **(B)** Screening Strategy 2 was initiated by 3-fold selection of peptide phage for their ability to bind to the TM. **(C)** The resultant TM-binding library was then screened for the ability to penetrate into the TM. **(D)** Finally, the TM-penetrating library was screened for the ability to cross the TM into the ME. EC, external canal.

In the first strategy, illustrated in Figures 1A–D, successive selection of an unselected library to a TM-binding library followed by a TM-penetrating library and finally a TM-transiting library was employed. Each of the phage clones and corresponding exogenous peptide sequences was given a sequential name with a BPT acronym (Binding-Penetrating-Transiting).

For the second strategy, the unselected library was applied to the TMs. The ME contents were harvested carefully and used to infect *E. coli*. for library phage amplification. This process was repeated twice, and then in the final round, 30 phage clones were selected and their inserts were sequenced. Again, the phage clones were given sequential names with a TMT acronym (TM transmigrating), respectively.

**Figure 2:**
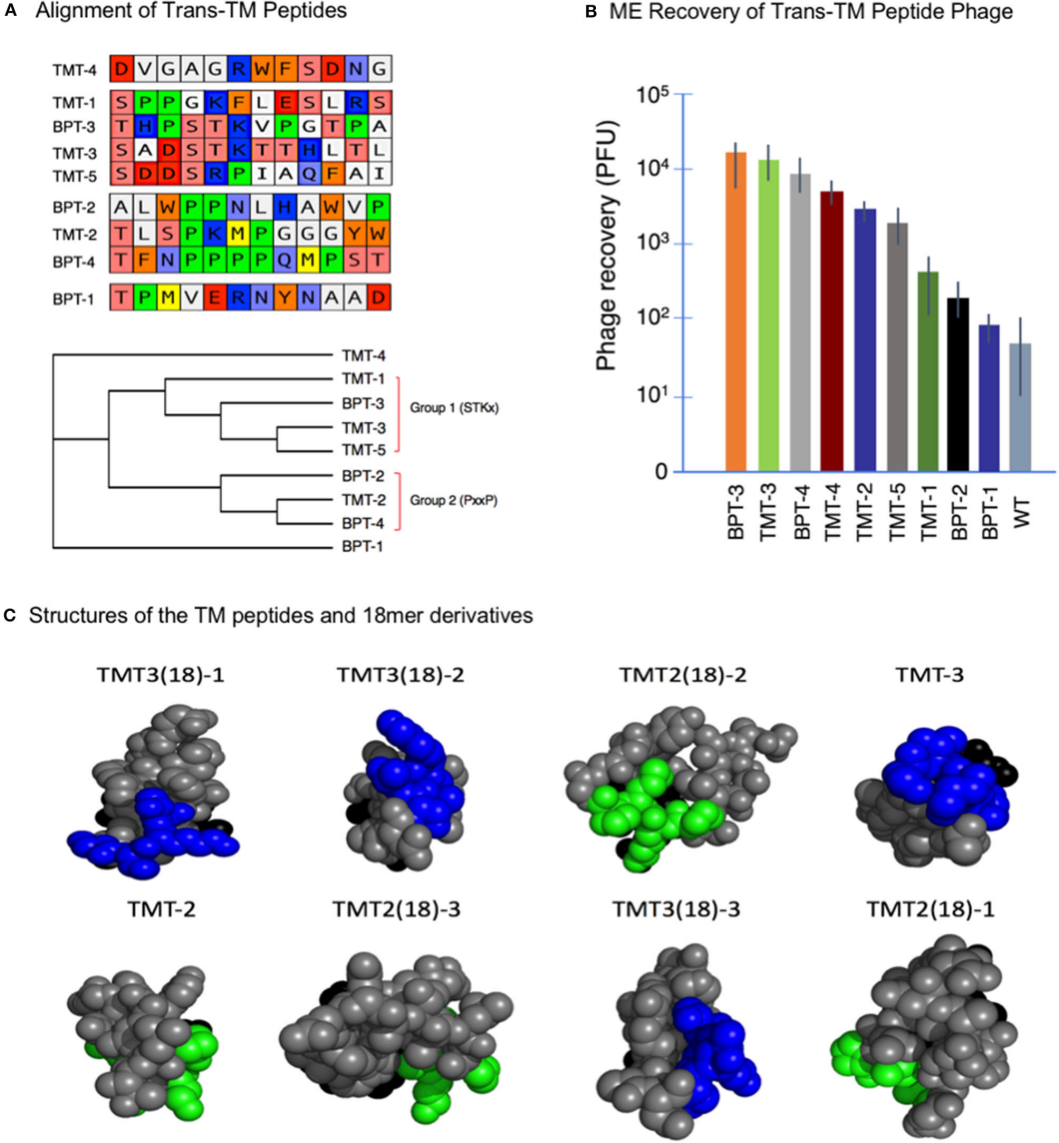
**(A)** Alignment of nine trans-TM 12-mer peptides. The peptides aligned into two groups characterized by the presence of two different motifs. Two peptides did not align but appeared more similar to the STKx motif group. **(B)** Recovery of nine trans-TM phage from the ME, after a 1-h incubation on the exterior surface of the TM. A wide range of recovery was observed, indicating differential rates of transport [Data from Kurabi et al. (25)]. **(C)** All-atom sphere structural representations of the 18-mer and parent 12-mer TM-transiting peptides from the optimization study (see Table 1) as predicted by PEPFOLD- 3.0. The terminal six amino acids of the pIII peptide were included in the structure. The predicted structures are illustrated in head-on view, from the free end of the peptide, with the pIII amino acids at the far side of the predicted structure and hidden from view. The consensus motifs shared by the different peptides ST(K/R) and PxxP motifs are indicated in blue and green, respectively. The peptides are arranged in descending order of ME recovery and thus of transport rate. It is clear from the figure that for peptides with highest levels of transport, the conserved motifs are prominently displayed on the free end of the peptide. This location presumably allows for better accessibility to binding sites on the TM [Data from Kurabi et al. (26)].

Strategy 1 yielded four unique peptide phages that were present more than once in the 30-phage sequence sample, suggesting that they were most efficient in crossing the TM, labeled BPT1-4. Strategy 2 led to the isolation of five unique peptide phages in all, labeled TMT1-5. The nine peptide sequences from both strategies were aligned using the MacVector software, as shown in Figure 2A. Four peptides aligned into one group characterized by all or part of an STKx consensus motif. Three peptides aligned into a second group showing all or part of a PxxP consensus motif. The remaining two peptides aligned separately, but they appeared to be more closely related to the STKx motif group than the PxxP motif group.

These nine phages were then amplified individually to confirm selectivity and compare transport rates. The results are illustrated in Figure 2B, which shows the ME recovery of the top 9 peptide phage after a 1-h exposure on the exterior surface of the intact TM. It is clear from the figure that all peptide-bearing phages were recovered at rates greater than that seen for the WT phage. These results provide evidence for the successful selection and effective enrichment of phage clones that cross the TM. It is also clear that phages bearing different exogenous peptides crossed the TM with variable efficiency.

### Trans-TM Phage Transport Is an Active Process

In order to learn more about the mechanisms of phage transport phenomena, several experiments were designed to evaluate the impact of concentration, temperature, and oxygen levels on transport across the TM. Increasing the concentrations of phage applied over a fixed time period resulted in proportionally increased ME phage recovery. Next, we tested the sensitivity of transport to oxygen deprivation and temperature using the *ex vivo*, intact ME bulla. When ME recovery of phage TMT-3 was assessed at various times after death, transport was observed up to 6 h postmortem, after which it ceased. When the bulla temperature was lowered to 4°C, transport over 1 h was almost entirely eliminated. These results suggest that transport across the intact TM occurs via an active, as opposed to a passive, mechanism.

### Additional Amino Acids Can Increase Transport

It seemed possible that 12 amino acids are not sufficient to provide an optimal peptide structure for interaction with the transport mechanisms of the TM. To explore this possibility, we chose two 12-mer trans-TM peptides with different transport rates, TMT-2 and TMT-3, and constructed two new phage libraries for each by adding six amino acids at random to the carboxy terminus, corresponding to the free end of the peptide (26). The two libraries were then screened using a brief (15 min) exposure to the exterior surface of the TM for three rounds followed by sequencing. Quantification of the transport of selected 18-mer peptide phage from each library revealed some with higher transit rates. For the 12-mer peptide with lower transport rate, TMT-2, the best 18-mer showed a doubling of ME recovery. However, for the 12-mer with the higher transport rate (TMT-3), the best 18-mer showed an 8-fold increase in transport (Table 1).

**Table 1:** The sequences and ME recovery of the trans-TM, 12-mer backbone peptides (TMT-2 and TMT-3) plus the top 18-mer peptide phages from the 18-mer screen.

**Transport category**	**Name**	**Sequence**	**Mean recovery from ME (PFUs)**	**Standard error**
Highest	TMT-3(18)-1	EGHLFPSADSTKTTHLTL	90,000	30,000
	TMT-3(18)-2	KISVYRSADSTKTTHLTL	40,000	10,000
	TMT-2(18)-2	SPTIFSTLSPKMPGGGYW	12,000	7,000
	TMT-3	*SADSTKTTHLTL*	10,800	3,000
	TMT-2	*TLSPKMPGGGYW*	5,900	1,310
	TMT-2(18)-3	VPQGATTLSPKMPGGGYW	3,000	1,080
	TMT-3(18)-3	TEVTSRSADSTKTTHLTL	280	100
Lowest	TMT-2(18)-1	LANNNLTLSPKMPGGGYW	120	100

*Incubation was 1 h on the exterior surface of the TM. A wide range of recovery was observed, indicating differential rates of transport [Data from Kurabi et al. (26)]*.

### Trans-TM Peptide Phages Do Not Cause Acute Middle or Inner Ear Damage

To evaluate the effects of phage on the middle and inner ears, we applied peptide-bearing or WT phage particles in saline into the normal rat ME for 1, 2, or 3 days via surgical transbullar injection. Saline injection was an additional control. We evaluated inner ear function by auditory brainstem response (ABR) pre-operatively and at 1, 2, and 3 days post ME injection. Responses to stimuli for 8, 16, and 32 kHz were obtained at intensity levels descending from 90 to 5 dB SPL. The pure tone ABR thresholds were increased by the same amount in all three treatment groups at day 1, presumably due to fluid being present in the ME. However, thresholds recovered to pre-operative levels in all groups by day 3 (27). Despite the high levels of phage applied to the ME, titers of perilymph indicated that phage transport into the inner ear was minimal. This represents that using phage particles is safe as a drug delivery technique.

### Peptides Mediate Transport Across the TMs of Other Mammalian Species, Including Human

The ability of peptides to mediate the transport of bacteriophage, which at nearly 1 μm in length are very large particles, suggests that they might be able to support the non-invasive transport of other large cargo, including drugs, drug particles, or gene therapy vectors across the intact TM and into the ME. This would only be useful, however, if transport was possible across the TM of patients. We therefore developed an *in vitro* assay consisting of an upper chamber into which fluid with phage could be introduced and a lower chamber for phage recovery. A silicone rubber membrane with an appropriately sized opening was used to seal a TM fragment in between the two chambers. The diameter of the two chambers was 1.5 mm, small enough that TM fragments could be sealed. Dye was included in the upper chamber to detect leaks, and the device was also tested with WT phage to determine that non-transporting phages were excluded from the lower chamber (28).

We first tested trans-TM phage TMT-3 transport across TMs from NTHi-infected MEs of rats as well as from rats with normal MEs. There was no difference in transport. We then tested TM fragments from uninfected rat, guinea pig, and rabbit MEs and found that transport for all three species was similar. Finally, we obtained discarded TM fragments from human patients undergoing TM repair and determined that the transport occurred at comparable levels across the human TM as well (28).

### Trans-TM Peptides Can Cross the TM Independent of Phage

It was not clear from our results whether peptides mediated transport across the TM, or whether attachment and the display of peptides by phage was necessary. To investigate this, we linked a DNA oligomer, to serve as a PCR template, to a trans-TM peptide TMT-3 (SADSTKTTHL). The oligo was 150-bp in length and cross-linked to the free peptide (Biosynthesis, TX, USA). We used our *in vitro* model since the infected *in vivo* ME would likely contain DNases that might degrade the DNA-tag. The level of DNA-tag was quantified in fluid from the lower chamber of the assay described above, by qPCR. Interestingly, we found that transport of peptide linked to DNA occurred at a comparable rate to that of peptide linked to phage (28).

### Transport Rate Is Related to Predicted Peptide Structure

The peptides identified by phage display screens were found to contain one of two common motifs: ST(K/R)T or PxxP. It was hypothesized that these motifs were related to the transport characteristics of the peptide phage. The structures of the two 12-mer and six 18-mer peptides discussed above were therefore analyzed. Predicted peptide structures, as expressed on the end of the pIII phage protein, were generated using the PEPFOLD program (29). The structures were then compared to the observed recovery of peptide phage from the ME after a standard TM exposure (Table 1). It was found that expression of either of the above motifs at the free end of the peptide, farthest from the amino terminus of the phage pIII protein, was associated with the highest phage recovery (Figure 2C) and, thus, with the highest efficiency of transport.

## Discussion

### Potential for Non-invasive Therapeutic Delivery

Phage display technology is a well-established method that has been successfully applied in novel drug discovery methods. Because the peptides discovered in our studies can mediate the transport of large phage particles through the TM, they also have the potential for non-invasive delivery of large drug packages to the ME. Moreover, the ability of DNA-tagged peptide alone to transit the TM at an equivalent rate to peptide-phage suggests that these peptides can be used for delivery of gene therapy DNA constructs. This is especially true since most gene therapy vectors are modified viruses. Since peptide-expressing bacteriophages are also modified viruses, we have already established a proof-of-principal for trans-TM delivery for viral vectors. Many genes with mutations that contribute to OM susceptibility have been discovered [e.g., (30, 31)]. A number of these, especially those that affect innate immunity [e.g., (16)], would be appropriate targets for gene therapy. However, which target(s) would be the most appropriate remains an open question.

Local drug delivery approaches into the inner ear such as intratympanic and intracochlear drug application methods, which limit systemic exposure, have become accepted as tools to manage hearing disorders. The biological bases for such treatments has been indicated by recent research [reviewed in (32)]. The injection of steroids into the middle ear has recently become a treatment of choice for Meniere's disease and sudden hearing loss (33). Steroids have also been shown in animal models to ameliorate the effects of noise trauma (34) and ototoxicity (35). Similarly, while the restoration of hearing by HC regeneration has yet to be achieved in humans, extensive experimental evidence suggests that this will be possible in the future using gene therapies (36). Recently, there has been tremendous progress in the development of gene therapy vectors to treat sensorineural hearing loss in animal models *in vivo* (37–39). Access of viral vectors to the cochlea employing the round window membrane (RWM) route enables this rapid and direct application. However, a major impediment to current and future medical treatments for hearing loss is the issue of drug delivery to the inner ear. Significant hurdles remain before such technologies can be translated toward clinical use. These include addressing engineering more specific and effective inner ear delivery vehicles, enhancing permeability of RWM to viral vectors, delivery across the blood-labyrinth barrier, improving surgical access, and validating specific targets (36, 40).

Currently direct access to the RWM is achieved through an intratympanic surgical procedure where a small perforation in the TM is created to apply the therapeutic drug into the middle ear. Alternatively, some drugs, such as steroids, will penetrate the RWM by diffusion. However, larger drugs and gene therapy vectors cannot easily transit the RWM. The existence of peptide-mediated active transport of cargo across the TM raises the question of whether a similar mechanism might be present in the RWM. If so, injection of peptide-labeled treatments into the middle ear would provide ready access to the inner ear. It is also possible that peptides targeting the intact TM and intact RWM could both be linked to drugs or viral vectors and mediate transport from the external to the inner ear. We are currently screening a phage peptide library to determine whether RWM-penetrating peptides that can carry cargo can be identified.

## Conclusions

Phage display, used to screen 10 billion random 12-mer peptides, identified several rare peptides that could mediate transport from the external to the middle ear, across the intact TM. Trans-TM transport is active, related to peptide structure, and occurs in several mammalian species including humans.

These peptides are able to mediate the transport of M13 bacteriophage, which are nearly 1 μm in length, across the various layers of the TM. This includes the two epithelia of opposite orientation and the intervening connective tissue layer. It seems likely that this cargo capacity can be harnessed for the non-invasive delivery of large drug packages and gene therapy vectors.

### Future Directions

In order to determine whether drug and gene therapy delivery are feasible, additional work needs to be accomplished. While it is clear that trans-TM peptides can carry large cargoes such as phage, it needs to be shown that they are capable to transporting pharmacologically meaningful quantities of drugs and/or effective gene therapy. Only then would they be useful for the treatment of patients. It is also necessary to identify the mechanism of transport, since utilizing an uncharacterized mechanism in patients might be judged as too risky. Whether trans-TM peptides can be utilized long-term without damage to the middle and inner ears needs to be determined. It is also possible that the peptides discovered in rats are not optimal for transport in humans. The *in vitro* assay that we developed to test transport across the human TM could be used to screen a phage library using human TM fragments. This could identify peptides with higher rates of transport across the human membrane.

## Author Contributions

All authors participated in the scientific research reviewed in the article and reviewed and approved the manuscript and figures.

## Conflict of Interest

AR is a co-founder of Otonomy Inc., serves as a member of the Scientific Advisory Board, and holds an equity position in the company. The UCSD Committee on Conflict of Interest has approved this relationship. Otonomy, Inc. played no part in the research reported here. The remaining authors declare that the research was conducted in the absence of any commercial or financial relationships that could be construed as a potential conflict of interest.

## Supplementary Material

Supplementary Figure 1 Structure of the ear showing the different principal parts: external ear canal, eardrum (tympanic membrane), the middle ear space with its ossicles, and inner ear (cochlea).
