# Supplementary figures and images for: Molecular Screening Strategy to Identify a Non-invasive Delivery Mechanism for the Treatment of Middle Ear Disorders
Source: Front Med (Lausanne). 2020 Dec 18;7:503819. doi: 10.3389/fmed.2020.503819 (PMC7775502; doi:10.3389/fmed.2020.503819)

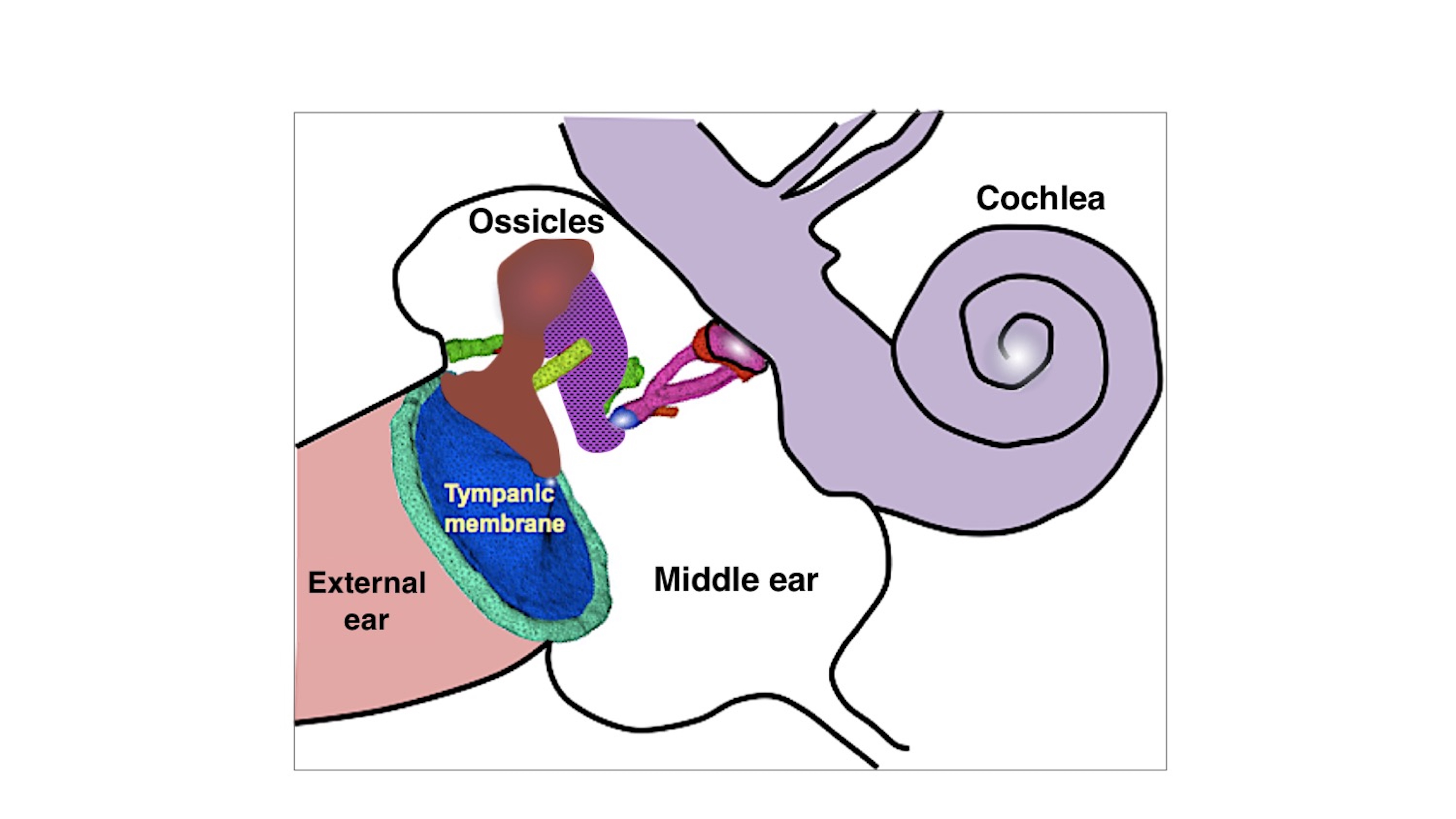

Supplement: Supplementary Figure 1 — Structure of the ear showing the different principal parts: external ear canal, eardrum (tympanic membrane), the middle ear space with its ossicles, and inner ear (cochlea). [file Image_1.jpg]
